# Verification of autoclaving-cooling treatment to increase the resistant starch contents in food starches based on meta-analysis result

**DOI:** 10.3389/fnut.2022.904700

**Published:** 2022-07-19

**Authors:** Didah Nur Faridah, Rhoito Frista Silitonga, Dias Indrasti, Frendy Ahmad Afandi, Anuraga Jayanegara, Maria Putri Anugerah

**Affiliations:** ^1^Department of Food Science and Technology, Faculty of Agricultural Engineering Technology, IPB University, Bogor, Indonesia; ^2^Southeast Asia Food and Agricultural Science and Technology (SEAFAST) Center, Lembaga Penelitian dan Pengabdian Kepada Masyarakat, Institut Pertanian Bogor University, Bogor, Indonesia; ^3^Center for Agro-Based Industry, Ministry of Industry, Bogor, Indonesia; ^4^Deputy Ministry for Food and Agribusiness, Coordinating Ministry for Economic Affairs Republic of Indonesia, Jakarta, Indonesia; ^5^Department of Nutrition and Feed Technology, Faculty of Animal Science, Institut Pertanian Bogor University, Bogor, Indonesia

**Keywords:** autoclaving-cooling, cereal, meta-analysis, resistant starch, starch modification

## Abstract

Autoclaving-cooling is a common starch modification method to increase the resistant starch (RS) content. The effect of this method varies depending on the type of crop and treatment condition used. The objectives of this study were to verify the autoclaving-cooling treatment based on a meta-analysis result and to evaluate the physicochemical properties of modified starches. The meta-analysis study used 10 articles from a total of 1,293 that were retrieved using the PRISMA approach. Meta-analysis showed that the optimal treatments of autoclaving-cooling process that increase the RS content significantly, was in starch samples from the cereal group (corn, oats, rice) (SMD: 19.60; 95% CI: 9.56–29.64; *p* < 0.001), with water ratio 1:4 (SMD: 13.69; 95% CI: 5.50–21.87; *p* < 0.001), using two cycles of autoclaving-cooling (SMD: 16.33; 95% CI: 6.98–25.67; *p* < 0.001) and 30 min of autoclaving heating (SMD: 12.97; 95% CI: 1.97–23.97; *p* < 0.001) at 121°C (SMD: 12.18; 95% CI: 1.88–22.47; *p* < 0.001). Verification using corn flour and corn starch showed a significant increase in RS contents from 15.84 to 27.78% and from 15.27 to 32.53%, respectively, and a significant decrease in starch digestibility from 67.02 to 35.74% and from 76.15 to 28.09%, respectively. Treated sample also showed the pasting profile that was stable under heating and stirring.

## Introduction

Resistant starch (RS) are any starch fractions that cannot be absorbed in the small intestine but can be fermented in the large intestine (1). It is recognized to impact gut bacteria, act as pre- and probiotics, has a role in preventing colon cancer, and help to overcome gastrointestinal dysfunction (2). RS is known to have a hypoglycemic effect, which can lower fasting blood glucose levels, increase insulin secretion and improve insulin sensitivity (3). According to Kumar et al. (4) and Lemlioglu-Austin et al. (5), the higher the content of RS in the sample, the lower the glycaemic index value produced. RS is classified into 5 types (RS 1, RS2, RS3, RS4, and RS5) with RS3 being the RS generated by retrograded amylose (2, 3). Various types of RS have been produced commercially, including Hi-maze^®^ whole grain corn flour (RS1 and RS2), Hi-maze 260 corn starch (RS2), Fibersym^®^ RW (RS4 Resistant Wheat Starch), and Novelose^®^ 330 (RS3).

A modification process can increase the amount of RS content in a material, and autoclaving-cooling is one of the methods that is widely used. This modification does not use chemicals, so it does not produce by-products in the final product. However, this modification process requires equipment such as an autoclave that can provide high temperature and pressure. It involves heating a starch sample suspended in water in an autoclave, then cooling it to produce retrograded amylose (6). The application of higher temperature along with higher pressure causes faster retrogradation rate, while cooling temperature leads to better retrogradation. The technical conditions used affect the gelatinization and retrogradation processes, which subsequently influence the amount of RS produced (7). In autoclaving-cooling, RS can be increased by adjusting processing conditions such as source of sample, heating and cooling temperature and time, also number of heating-cooling cycles (8). One example of a commercial RS product produced through a retrogradation process is Novelose^®^ 330.

Several studies stated that the more cycles used, the higher the RS content increase (1, 9–11). However, Ratnaningsih et al. (12) reported that autoclaving-cooling 1 cycle resulted in higher RS content than autoclaving-cooling with 3 cycles and 5 cycles. Rahmawati et al. (13) reported that the addition of cycles in the autoclaving-cooling process did not significantly affect the content of RS. Various studies have also been conducted to investigate differences in the use of heating time during the autoclaving process. Variations of autoclaving time used include 15, 30, 60, and 120 min. These variations have different effects on the of RS content (14–16).

The findings of studies did not clearly describe the effect of autoclaving-cooling treatment on RS contents. Therefore, a meta-analysis study needs to be carried out to evaluate the effect of the treatment used. Meta-analysis is a quantitative scientific synthesis of various research results that have been used in many scientific fields. Meta-analysis aids in the practice of evidence-based research and the resolution of conflicting study findings. The meta-analysis extracts one or more study outcomes in terms of effect sizes. The purpose of effect sizes is to put the results of a large number of research on the same scale by employing a number of metrics such as oddity and risk ratios, standardized mean differences, transformed correlation coefficients and logarithmic response ratios (17, 18). The effectiveness of hydrothermal starch modification such as heat moisture treatment (HMT) and annealing has been conducted (19, 20). However, the effectiveness of autoclaving-cooling modification and its physicochemical properties of the modified starch has not been done. The objectives of this study were to verify the autoclaving-cooling treatment based on a meta-analysis result and to evaluate the physicochemical properties of modified starches. This study provides the optimal treatment of the autoclaving-cooling process to achieve a significant increase in RS content, especially RS 3, which can be applied as functional food ingredients.

## Materials and methods

### Materials

Materials for meta-analysis study were articles obtained from several reputable online journal databases and published from 2000 to 2020. Corn flour and corn starch for verification are obtained from commercial products in Indonesia, under the brands “Mugo Tepung Jagung” and “Maizena 328,” respectively.

### Meta-analysis study

The studies were retrieved using PRISMA (Preferred Reporting Items for Systematic Reviews and Meta-Analysis) statement guidelines (21). Several reputable online journal databases, such as ProQuest, Science Direct, PubMed, Wiley Online Library, and Google Scholar were used to search and identify studies. Studies were limited to articles published from 2000 to 2020 and searched with appropriate keywords, for instance “autoclaving cooling starch,” “autoclaving cooling resistant starch” and “autoclaving cooling modification starch.” In order to identify relevant articles, keywords were combined with Boolean operators and advanced search tools.

Based on inclusion and exclusion criteria, studies were selected through screening and eligibility stages. Research articles from international journals indexed by Scopus (Q1–Q3) and web of science published from 2000 to 2020 were used as inclusion criteria. Articles also have to include sufficient data, namely source of starch, the value of RS from native (as control) and from autoclaving-cooling process (as experiment), standard deviation or standard error, and number of replications. Exclusion criteria included studies from books or patents, studies with raw materials other than starch, and studies with other treatments or multiple modifications.

Data were extracted and inputted into a worksheet developed by Afandi et al. (22) using the calculation +formula from Palupi et al. (23) and Borenstein et al. (17). The following information was gathered: source of starch, water ratio, number of autoclaving-cooling cycles, heating time, temperature of heating and cooling, mean and standard deviation of RS (control and experiment), and number of replications.

### Verification and characterization of physicochemical properties

Verification of the autoclaving-cooling treatments was carried out based on the results of the meta-analysis. Sample (fineness 100 mesh) was weighed as much as 60 g, then suspended with distilled water in a ratio (sample:water, 1:4). The sample was put into an autoclave for 30 min at a temperature of 121°C. The sample then continued was cooled in a refrigerator at 4°C for 24 h. The autoclaving-cooling was repeated once again, so that the total cycle is two times. The samples obtained were then dried in an oven at 50°C for 24 h. After that, the sample was mashed and shieved (100 mesh) for further characterization. Native and modified samples were analyzed for moisture, ash, protein and fat content according to AOAC (24). The RS content (25), starch digestibility (26) and gelatinization profile (using Rapid Visco Analyzer) of the samples were also determined. Starch morphology was observed using a light microscope, polarizing microscope and scanning electron microscopy (SEM) (27) while the crystalline analysis was carried out using Fourier Transform Infrared (FTIR) (28, 29).

### Statistical analysis

The meta-analysis data were analyzed by the effect size value using Hedges’d (standardized mean difference/SMD), with 95% CI (confidence interval) pooled through a random-effects model (22, 23) to assess the effect of autoclaving-cooling on RS content. A forest plot was used to evaluate the individual study and pooled effect sizes. The value of CI that did not include zero determined the significance of the autoclaving-cooling effect (30). Heterogeneity test was assessed using the *I*^2^ value (31), while publication bias was determined by funnel plot and Egger’s regression test. The subgroup analysis of crop type, water ratio, number of cycles, heating time and temperature of autoclaving were performed to analyze the subset of included studies. The meta-analysis process was performed using Meta-Essentials 1.5 software (32). The characterization data was analyzed by comparison of the native and treated sample using the paired *t*-test. The analysis was carried out at a significance level of 5% (95% CI) using SPSS 26 software.

## Results and discussion

### Autoclaving-cooling treatments to increase the resistant starch content based on meta-analysis

#### Included study analysis

**FIGURE 1:**
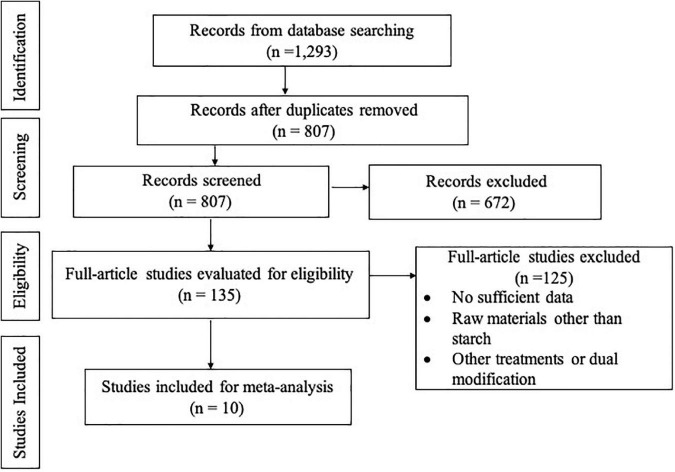
Schematic representation (PRISMA flow chart) of the literature review process.

A total of 1,293 studies were initially retrieved and screened by the search strategy. Then, 135 full articles were evaluated, with 125 studies being excluded, leaving 10 studies for meta-analysis (Figure 1). These studies were excluded for several reasons, including studies published in journals not indexed Scopus, as well as studies that did not have the required completeness of data, such as number of replication, standard deviation values, or data treatment (total cycle, temperature of autoclaving, and temperature of cooling) The required data information was extracted from the selected studies, resulting in 21 data (Table 1).

**TABLE 1:** Characteristic of included studies.

No	Study	Source of starch	Water ratio	AC cycle	Heating time (minutes)	Temperature (°C)		*n*	RS (% w/w)		Change of RS (%)
						Heating	Cooling		*C* (X ± SD)	*E* (X ± SD)	
1	Shah et al. (15)	Rice SR-1	1:4	2	30	121	4	3	4.42 ± 0.88	30.31 ± 0.79	585.75
2	Shah et al. (15)	Rice SR-2	1:4	2	30	121	4	3	8.26 ± 0.86	35.80 ± 0.90	333.41
3	Shah et al. (15)	Rice Pusa	1:4	2	30	121	4	3	5.91 ± 0.61	32.56 ± 0.63	450.93
4	Shah et al. (15)	Rice Jhelum	1:4	2	30	121	4	3	10.94 ± 0.59	38.65 ± 0.82	253.29
5	Sankhon et al. (36)	Parkia seed	1:4	4	120	110	5	3	28.96 ± 2.00	43.06 ± 2.00	48.69
6	Astuti et al. (37)	Arrowroot	1:5	3	15	121	4	3	22.56 ± 0.43	24.21 ± 2.14	7.31
7	Kim et al. (38)	Corn	1:3.5	4	60	121	4	3	0.30 ± 0.00	12.20 ± 0.30	3966.67
8	Giuberti et al. (39)	Sorghum grain	1:4	2	30	121	4	3	43.70 ± 5.89	56.90 ± 5.89	30.21
9	Shah et al. (15)	Oat Sabzaar	1:4	2	30	121	4	3	23.90 ± 1.27	38.88 ± 1.00	62.68
10	Shah et al. (15)	Oat SK020	1:4	2	30	121	4	3	17.39 ± 0.57	29.14 ± 0.61	67.57
11	Shah et al. (15)	Oat S090	1:4	2	30	121	4	3	17.14 ± 0.06	25.81 ± 1.04	50.58
12	Kasote et al. (40)	Pigeon pea	1:5	1	60	120	4	3	16.86 ± 0.26	3.96 ± 0.15	–76.51
13	Kasote et al. (40)	Green gram	1:5	1	60	120	4	3	11.60 ± 2.42	3.16 ± 0.16	–72.76
14	Kasote et al. (40)	Black gram	1:5	1	60	120	4	3	11.35 ± 0.77	4.07 ± 0.15	–64.14
15	Simons et al. (16)	Corn	1:5	1	15	110	4	2	0.12 ± 0.08	8.38 ± 0.11	6883.33
16	Simons et al. (16)	Great northern bean	1:5	1	15	110	4	2	40.83 ± 3.11	18.33 ± 0.67	–55.11
17	Simons et al. (16)	Pinto bean	1:5	1	15	110	4	2	43.86 ± 1.56	17.13 ± 0.59	–60.94
18	Simons et al. (16)	Black bean	1:5	1	15	110	4	2	30.52 ± 4.62	14.86 ± 0.41	–51.31
19	Simons et al. (16)	Lima bean	1:5	1	15	110	4	2	60.28 ± 0.64	14.88 ± 0.29	–75.32
20	Polesi and Sarmento (41)	Chickpea	1:10	1	30	121	4	3	31.87 ± 1.35	16.35 ± 0.86	–48.70
21	Aparicio-Saguilán et al. (42)	Banana	1:3.5	3	60	121	4	3	1.51 ± 0.10	16.02 ± 0.24	960.93

n, number of replications; RS, resistant starch; X, mean; SD, standard deviation; C, control (native); E, experiment (treated starch).

In autoclaving-cooling process, heating above the gelatinization temperature resulted in the dissociation of hydrogen bonds from the double helix structure of amylopectin, melting of crystallites and the release of the amylose fraction from the granules (7, 8, 33). The amylose fraction then binds to form a double helix structure and binds to other double helix structures to form crystallite, so that amylose recrystallizes and forms RS3 (34).

**FIGURE 2:**
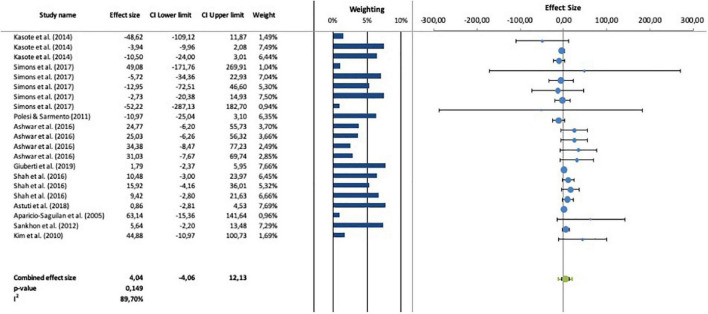
Forest plot between individual studies to analyze the effect of RS content by autoclaving-cooling process.

The forest plot of individual studies was shown in Figure 2. The CI value from combined effect size (SMD 4.04; 95% CI: –4.06 to 12.13; *p* = 0.149) included zero. It is revealed that single modification by autoclaving-cooling process statistically had no significant effect on increasing the RS content. This insignificant outcome may be due to the numerous variations used in the autoclaving-cooling process.

The heterogeneity (*I*^2^) among studies is 89.70% and classified as high heterogeneity (greater than 75%) (31). The starch source of crop and treatment conditions (such as number of autoclaving cycles and heating time) can contribute to this high heterogeneity value of RS change (35). To evaluate the effect of these variants on changes of RS, the subgroup analysis was performed. Subgroup analysis was conducted to groups with *n* ≥ 2.

#### Subgroup analysis based on type of crop

**FIGURE 3:**
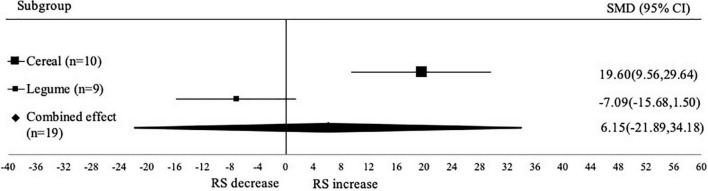
Forest plot from subgroup analysis based on type of crop. A positive value indicates the increase of RS content, while negative value indicates the decrease of RS.

Analysis of RS content based on type of crop consist of 2 types of crops, namely cereal and legume (Figure 3). The autoclaving-cooling treatment enhanced the RS content in cereal group (SMD: 19.60; 95% CI: 9.56–29.64; *p* < 0.001) but did not significantly affect the legume group (SMD: –7.09; 95% CI: –15.68 to 1.50; *p* < 0.001). The amounts of RS are closely linked to the levels of amylopectin and amylose contained in a substance. In general, the higher amylose/amylopectin ratio, the more RS is present. This is because the chain structure of amylose is small and easy to orient and regenerate, while amylopectin has a dendritic structure and is difficult to orient (43).

Figure 3 also showed a negative value (decrease of RS) from the legume group. This decrease may be related to the destruction of RS1 and RS2 during the autoclave process, where the amount of RS1 and RS2 degraded was more than the RS3 formed by an incomplete starch retrogradation (44). The destruction of RS1 and RS2 in a more open structure with fragmented starch chains of varying lengths. After gelatinization, these flexible linear amylose molecules align themselves into tight linear configurations, forming helices, rendering the α-1,4 glycosidic linkages inaccessible to amylase (45).

**FIGURE 4:**
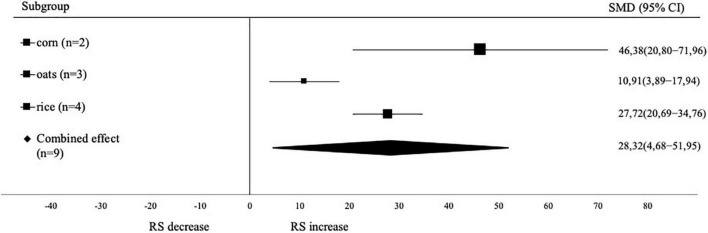
Forest plot from subgroup analysis based on cereal sample. A positive value indicates the increase of RS content, while negative value indicates the decrease of RS.

The subgroup of cereal sample types was investigated to determine which type of sample had the highest effect on increasing levels of RS for laboratory verification (Figure 4). There were three groups of samples analyzed, namely corn, oats and rice. Based on the results of the analysis, the sample groups of corn, oats and rice had a significant effect in increasing the levels of RS. The highest effect size value was corn (SMD 46.38; 95% CI: 20.80–71.96), followed by rice (SMD 27.72; 95% CI: 20.69–34.76) and oats (SMD 10.91; 95% CI: 3.89–17.94).

The higher effect in corn samples could be attributed to the higher amylose content when compared to rice and oat samples. The amylose content in normal corn is 30%, in oats is 28%, and in normal rice is 20–22% (46, 47). The higher the amylose content, the faster starch retrogrades, and increases the formation of RS (8). Punia et al. (48) also stated that the retrogradation rate of corn starch was higher than that of oat starch.

The rate of retrogradation is also affected by the unit-chain length distribution of amylopectin. Srichuwong et al. (27) stated that there is a positive correlation between the distribution of DP (degree of polymerization) 16–26 and the rate of retrogradation, while the DP 8–12 (short chain) has a negative correlation. The more composition of DP 16–26, the rate of retrogradation will be faster, conversely the more unit-chain DP 8–12, the rate of retrogradation will be slower. Corn starch and rice starch are known to have the unit-chain length distribution of DP 13–24 by 56.7 and 52.1%, and the unit-chain length distribution of DP 9–12 by 31.4 and 34.5%, respectively (27). The distribution of the unit-chain DP 13–24 in oat starch ranged from 54.78 to 57.83% while the DP 6–12 ranged from 27.53 to 32.07% (49).

#### Subgroup analysis based on water ratio

**FIGURE 5:**
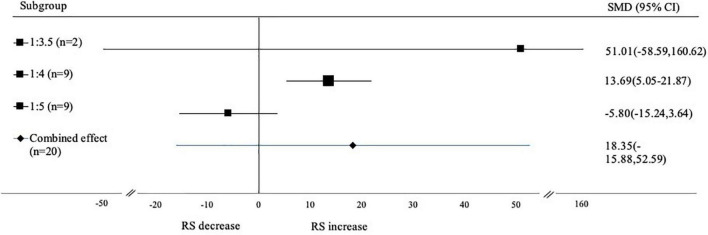
Forest plot from subgroup analysis based on water ratio. A positive value indicates the increase of RS content, while negative value indicates the decrease of RS.

Based on water ratio, the studies were sub grouped into 3 groups, namely the ratio of 1:3.5; 1:4 and 1:5 (Figure 5). The analysis results showed that ratio (sample:water) of 1:4 had a significant effect on increasing the RS content (SMD: 13.69; 95% CI: 5.50–21.87; *p* < 0.001), while the ratio 1:3.5 (SMD: 51.01; 95% CI: –58.59 to 160.62; *p* < 0.001) and 1:5 (SMD: –5.80; 95% CI: 15.24–3.64; *p* < 0.001) had no significant effect. The higher water content during gelatinization increases starch gelatinization and retrogradation enthalpy. This value is associated with the amount of single and double helical structures (50).

#### Subgroup analysis based on number of autoclaving-cooling cycles

**FIGURE 6:**
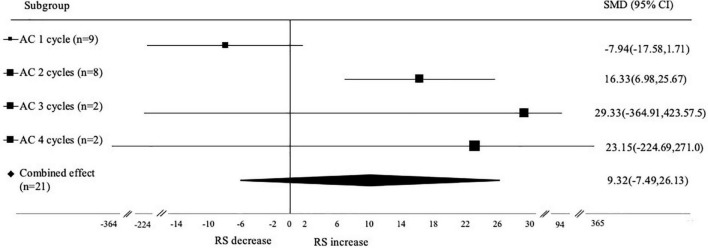
Subgroup analysis of autoclaving-cooling number of cycles. A positive value indicates the number of cycles that increase the RS content, while negative value indicates RS decrease.

Based on the number of autoclaving-cooling cycles, the studies were sub grouped into 4 groups (AC 1 cycle, 2 cycles, 3 cycles, and 4 cycles) and presented in the forest plot SMD, 95% CI (Figure 6). The analysis result showed that autoclaving-cooling 2 cycles had a significant effect on increasing the RS content (SMD: 16.33; 95% CI: 6.98–25.67; *p* < 0.001), while autoclaving-cooling 1 cycle (SMD: –7.94; 95% CI: –17.58 to 1.71; *p* < 0.001), 3 cycles (SMD: 29.33; 95% CI: –364.91 to 423.52; *p* < 0.001) and 4 cycles (SMD: 23.15; 95% CI: –224.69 to 271.0; *p* < 0.001) had no significant effect.

Many studies applied autoclaving-cooling starch modification with varying numbers of cycles. The addition of cycles is expected to increase the formation of short chain amylose fraction and increase the amount of retrograded amylose, resulting in more RS (51). Setiarto et al. (11) modified taro flour by autoclaving-cooling 1 cycle and 2 cycles. The RS produced by a 2 cycles process was increased 169.98%, higher than the RS produced by a single cycle process (91.77%). Likewise, the increasing RS content produced from 3 cycles autoclaving-cooling on purple water yam (48.92%), yellow water yam (75.86%) and white water yam (73.51%) were higher than increasing RS from 2 cycles (37.87%; 54.43%; 58.35%) and 1 cycle (7.5%; 27.09%; 4.03%), respectively (51). The number of autoclaving-cooling cycles, on the other hand, can increase the depolymerization of long-chain amylose into short-chain fractions (51, 52), as well as the breaking of amylose chains into simple sugars. The presence of these simple sugar components is known to slow down the retrogradation process, thereby affecting the formation of RS (53). Autoclaving-cooling cycle is closely related to retrogradation kinetics. The significance of the autoclaving-cooling 2 cycles could be influenced by its retrogradation kinetics that higher than in other cycles.

#### Subgroup analysis based on heating time

**FIGURE 7:**
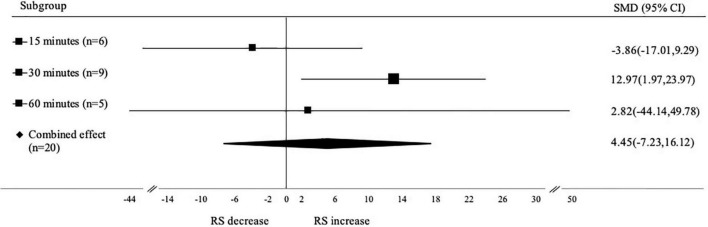
Subgroup analysis of autoclaving-cooling heating time. A positive value depicts the RS content increase, while negative value depicts the RS content decrease.

Analysis for subgroups based on heating time of autoclaving was shown in Figure 7. Heating time 30 min (SMD: 12.97; 95% CI: 1.97–23.97; *p* < 0.001) has a significant effect on increasing the RS content compared to heating time 60 min (SMD: 2.82; 95% CI: –44.14 to 49.78; *p* < 0.001). Meanwhile, heating time 15 min (SMD: –3.86; 95% CI: –17.01 to 9.29; *p* < 0.001) has no significant effect on decreasing the RS content. The application of heat to a certain temperature and time is required to confirm that the starch is gelatinized fully thus it will undergo retrogradation later.

#### Subgroup analysis based on autoclaving temperature

**FIGURE 8:**
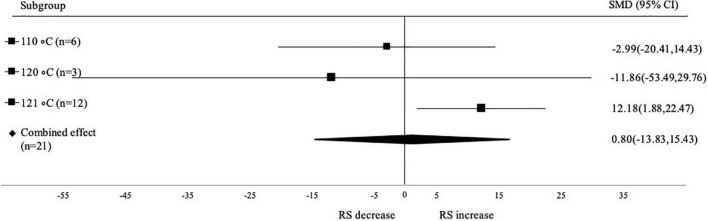
Subgroup analysis of autoclaving temperature.

Analysis based on temperature of autoclaving showed that autoclaving at 121°C has significant effect on increasing the RS content (SMD: 12.18; 95% CI: 1.88–22.47; *p* < 0.001) compared to autoclaving at 110°C (SMD: –2.99; 95% CI: –20.41 to 14.43; *p* < 0.001) and 120°C (SMD: –11.86; 95% CI: 53.49–29.76; *p* < 0.001) (Figure 8). Aside from the number of autoclaving-cooling cycles, the temperature in the autoclaving process influences the degree of retrogradation, which affects the RS content (54). The temperature of 121°C is higher than the temperature of traditional gelatinization. Therefore, it leads to better gelatinization. Gelatinization at lower temperatures might require a longer time period. Right combination of temperature and time is needed for retrogradation. The cooling temperature of the process was not analyzed further because almost all data used the cooling temperature at 4°C (only 1 data used 5°C).

#### Publication bias

**FIGURE 9:**
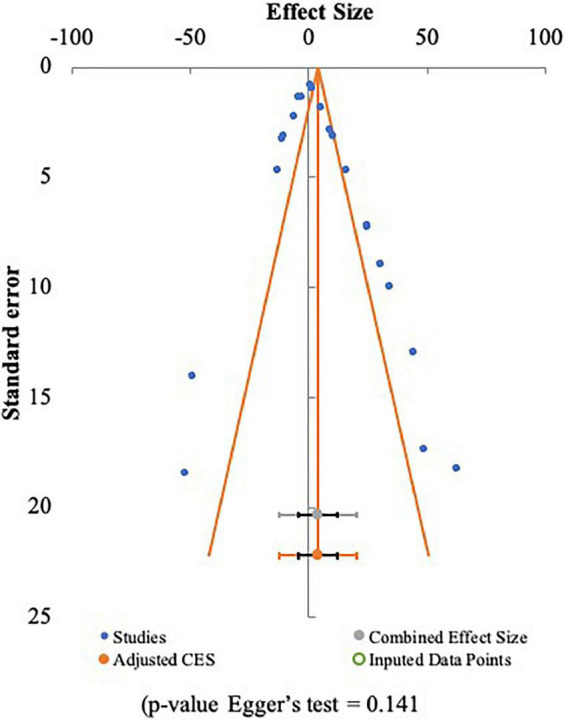
Publication bias analysis of included studies.

The funnel plot and Egger’s test were used to assess the publication bias of included studies, with *p* < 0.05 being statistically significant for publication bias (22). The publication bias analysis (Figure 9) showed a funnel plot with symmetric data points. It indicated that the data is not affected by bias (32, 55). It is also demonstrated by the results of the analysis using the Egger’s test where the *p*-value is greater than 0.05 and being statistically not significant for publication bias. This study has limitations, including the small sample size and heterogeneity among studies that may affect the reliability of the results. Larger sample sizes are needed to confirm the current results.

### Physicochemical properties

#### Chemical properties

Verification was carried out based on the results of the meta-analysis, namely using corn samples, which were treated by 2 cycle of autoclaving-cooling, with (sample:water) ratio 1: 4 and autoclaving for 30 min at 121°C. The samples used were in the form of starch and flour from corn. Corn flour is also used as a sample so that it can be used as a comparison for use in application, because corn flour is easier to obtain than corn starch which requires an extraction stage. The chemical properties of native and treated samples are presented in Table 2.

**TABLE 2:** Chemical properties of native and modified samples.

Parameter (% db)	Sample			
	Corn flour		Cornstarch	
	Native	Treated	Native	Treated
Moisture	12.72 ± 0.12	9.43 ± 0.04*	11.93 ± 0.12	11.63 ± 0.04*
Ash	0.64 ± 0.02	0.14 ± 0.01*	0.16 ± 0.01	0.16 ± 0.01
Protein	6.86 ± 0.19	7.32 ± 0.34*	0.41 ± 0.03	0.37 ± 0.06
Fat	2.56 ± 0.05	0.41 ± 0.01*	0.12 ± 0.01	0.04 ± 0.01*
Carbohydrate (by difference)	89.55 ± 1.48	92.13 ± 0.35*	99.31 ± 0.04	99.43 ± 0.06*
Amylose	32.47 ± 0.34	30.35 ± 0.67*	39.75 ± 0.53	38.37 ± 1.69
Resistant starch	15.84 ± 0.28	27.78 ± 1.28*	15.27 ± 3.79	32.53 ± 3.97*
Starch digestibility	67.02 ± 2.37	35.74 ± 2.67*	76.15 ± 6.24	28.09 ± 3.48*

Values expressed are mean ± standard deviation (number of replications = 4).

*There is significant difference between native and treated sample.

In corn flour, all parameters showed significant changes from native and treated samples, while in cornstarch, the ash content, protein content, and amylose content did not change significantly. The moisture of each sample is at a low value, which is below 12%. Low moisture is needed especially for samples in the form of flour, to increase the shelf life of the product. The ash content in the corn flour decreased from 0.6 to 0.14%, while in cornstarch, the ash content value did not change. The decrease in ash content in the sample after treatment indicates that the autoclaving-cooling process in this experiment resulted in a cleaner sample (56). Autoclaving decreases the number of low melting point during ashing. In flour samples the amount of volatile mineral elements is higher than that in starch samples. The protein content of cornstarch decreased from 0.41 to 0.31%. The decrease of protein after the autoclaving-cooling process also reportedly occurred due to the heating process at high temperatures during the autoclaving-cooling process which resulted in the protein structure being damaged (14, 42, 51). However, from Table 2, there was an increase in protein content in the corn flour after treatment from 6.86 to 7.32%. Similar results also occurred in the samples of potatoes (57) and black beans (44) from the autoclaving-cooling treatment, which showed an increase in protein content from 0.13 to 0.43% and from 7.54 to 13.47%. Denaturation leads to changes in the three-dimensional structure of protein. It happens during the application of thermal treatments. It causes the decrease of protein solubility due to aggregation and precipitation. This phenomenon resulted in the increase of protein content in modified samples (44). The autoclaving-cooling process was also seen to reduce the fat content of each sample. These results are in accordance with the results obtained by Aparicio-Saguilán et al. (42) and Rosida et al. (51).

Characterization of the samples was also carried out through analysis of amylose content, RS content and starch digestibility. In Table 2, there was a decrease in the amylose content of the corn flour, from 32.47 to 30.35%, while the amylose content of the cornstarch sample did not change significantly. A decrease in amylose levels was also seen in a study conducted by Shah et al. (15). In this research, the amylose content of the two cycles of autoclaving-cooling samples decreased in three types of oat samples, namely Sabzaar oats (from 26.97 to 25.91%), SKO20 oats (from 26.13 to 25, 43%) and SKO90 oats (from 25.81 to 25.45%). The decrease in amylose content in the sample after autoclaving-cooling treatment can occur due to hydrolysis of long-chain amylose molecules to short-chain molecules because of the gelatinization under pressure (52). This process could break the amylose chain into simple sugars, such as dextrin. The dextrin could not form blue complex with iodine during the amylose analysis therefore the observed amylose concentration was lower.

A significant increase in the levels of RS was seen in corn flour and cornstarch after treatment (Table 2). This result is in accordance with the results of the meta-analysis that the autoclaving-cooling process in corn samples can significantly increase the levels of RS. The RS content in the corn flour increased by 75.38% (from 15.84 to 27.78%) while in cornstarch increased by 113.03% (from 15.27 to 32, 53%). The increase in RS levels in corn flour was lower than in cornstarch. Flour consists of other components such as fiber, protein and lipid, therefore the retrogradation rate becomes slower. The fat content (especially free fatty acids) in starch is known to have the potential to form amylo-lipid complexes (RS type 5), while the presence of protein can interfere with the process of incorporating starch molecules (54).

The value of RS content in treated cornstarch (32.53%) was higher than in the corn flour (27.78%). This could be caused by the amylose content of the cornstarch (39.75%) which was higher than in the corn starch (32.47%). The higher the amylose content in a material, the higher the RS content produced (58–60). High amylose content in a material affects the formation of RS during the autoclaving-cooling process. The starch retrogradation process that occurs in autoclaving-cooling is mainly caused by amylose interactions, because hydrogen bonds between amylose are easily formed. The more amylose fractions that came out of the starch granules during the gelatinization process, resulted in the formation of more retrograded starch during the cooling process, thereby increasing the formation of RS (51). Changes in RS content are not only affected by amylose content, but associated with other factors, such as crystalline type, amylose:amylopectin ratio, length of amylopectin chain and autoclaving-cooling conditions. The RS content of the treated corn flour and cornstarch was categorized as very high, namely 27.78 and 32.53% (above 15%) (25). This value is close to the commercial retrogradation resistant starch (RS3) (Novelose 330), which is 37.0% (42).

The digestibility of starch in the treated corn flour decreased by 46.67% (from 67.02 to 35.74%), while the maize starch sample decreased by 63.11% (from 76.15 to 28.09%). The decrease in starch digestibility in the corn flour was also in line with the smaller increase in RS content when compared to the cornstarch. In autoclaving-cooling process, there is a rearrangement of starch molecules, both between amylose-amylose and amylose-amylopectin, so that it can strengthen starch bonds which make starch more difficult to digest (6). The amylose fraction which has a linear structure is also known to facilitate the formation of cross-links in the presence of hydrogen bonds, thus forming a more compact amylose structure that is difficult to hydrolyze by enzymes (42, 61). Apart from proportions and structure of amylose and amylopectin, the digestibility of starch also influenced by the starch granule (its morphology, surface features, molecular composition and supramolecular structures), proteins and lipids content. The presence of endogenous protein attached to the starch granule surfaces could reduce the granular swelling and restrict the access to digestive enzymes (62). The digestibility of starch from corn flour and cornstarch treated by autoclaving-cooling (35.74 and 28.09%, respectively) was lower than the digestibility of starch from commercial starch products Novelose 330 which was 33.33% (63). This proves that samples of corn flour and corn starch treated by autoclaving-cooling have the potential to be used as raw materials for functional food.

#### Crystallinity properties

Crystallinity properties were seen through changes in crystalline and amorphous regions in the starch structure before and after treatment through FTIR. FTIR can determine the level of helical arrangement of starch to see changes in starch due to gelatinization, retrogradation or storage processes. Polysaccharides in starch can be absorbed at a wave number of 800–1,200 cm^–1^, which is a fingerprint of the conformation and hydration of starch. Wave numbers 1,045–1,047 cm^–1^ and 1,020–122 cm^–1^ are bands for the crystalline region and amorphous region in starch granules. The ratio 1,045 cm^–1^/1,022 cm^–1^ indicates the arrangement of the crystalline regions, while the ratio 1,022 cm^–1^/995 cm^–1^ indicates the arrangement of the amorphous regions (29, 64). The changes in crystalline and amorphous regions from native and treated samples are presented in Table 3.

**TABLE 3:** Changes in crystalline and amorphous regions of corn flour and cornstarch based on FTIR analysis.

Sample	Crystalline	Amorphous
	A_1045_/A_1022_	A_1022_/A_995_
Corn flour native	0.9289 ± 0.0037	1.0249 ± 0.0007
Corn flour treated	1.0213 ± 0.0126	0.8759 ± 0.0062
Cornstarch native	0.9682 ± 0.0339	1.0302 ± 0.0030
Cornstarch treated	1.0376 ± 0.0023	0.8164 ± 0.0065

Values expressed are mean ± standard deviation (number of replications = 2).

Table 3 shows that there is an increase of the crystalline region and a decrease of the amorphous region in each of the treated samples. The crystalline region of corn flour native was 0.9289 and increased to 1.0213 due to the autoclaving-cooling treatment, as well as the amorphous region, which decreased from 1.0249 to 0.8759. Likewise, the native cornstarch experienced an increase in the crystalline region from 0.9682 to 1.0376 and a decrease in the amorphous region from 1.0302 to 0.8164. The ratio intensity of 1,045 cm^–1^/1,022 cm^–1^ also expresses the degree of order in starch (15). Increase of 1,045 cm^–1^/1,022 cm^–1^ ratio exhibits that crystallites at the granule surface of treated samples were better organized (39). This ratio intensity may differ depending on the modification treatment and the source of the sample. Maize starch treated with annealing increased the ratio intensity of 1,045 cm^–1^/1,022 cm^–1^, while heat-moisture treatment decreased the ratio (64).

Amylose content strongly affects the crystallization during retrogradation, which reduces the access of digestive enzymes. Thus increasing RS content (65). The application of autoclaving cooling was also observed to reorder double helices in the crystalline lamellae. This phenomenon is affected by amylopectin branch length as well. Longer amylopectin branch resulted in more packed double helices structure (66). It was also observed in annealing modification (67).

#### Morphology properties

**FIGURE 10:**
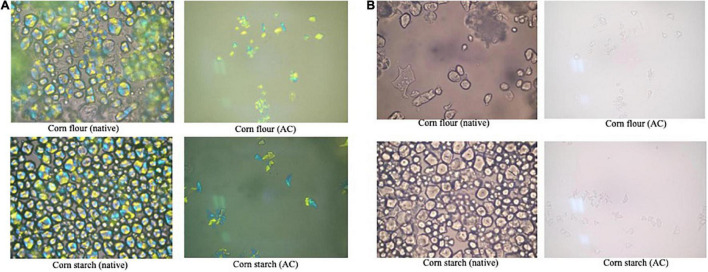
Morphological analysis of starch granules using a polarizing microscope (**A**) and a light microscope (**B**) (magnification × 1,000).

**FIGURE 11:**
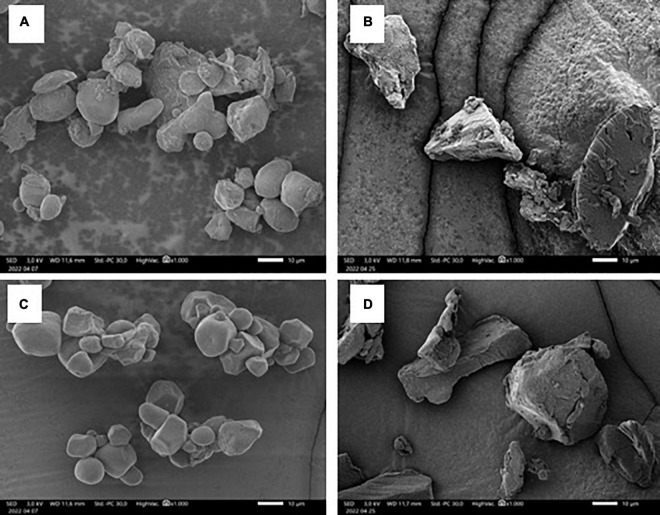
Scanning electron microscopy of native corn flour **(A)**, treated corn flour **(B)**, native cornstarch **(C)** and treated cornstarch **(D)** with 1,000 × magnification.

Morphological changes of starch granules were observed using a polarizing microscope and a light microscope (Figure 10) also using SEM (Figure 11).

Figure 10 showed that the native starch granules were still intact and had an oval shape. This indicates that the granule structure of the native starch has not been damaged in comparison to the treated starch. The native samples in Figure 10 also still exhibit birefringence and intact maltose cross pattern, as indicated by a clear blue-yellow color pattern. The presence of this birefringence indicates that the starch has not been gelatinized. Meanwhile, the birefringence properties of the treated starch were not observed.

Figure 11 showed that the autoclaving-cooling treatment causes the structure change of corn flour and cornstarch. This result is similar to that of Herawati et al. (68) and Faridah et al. (19), who performed an analysis on tacca starch and rice starch treated with autoclaving-cooling. The granules of treated samples become shapeless or irregular shapes. This irregular shape of crystalline was formed because of granules being damaged and swollen during the gelatinization and retrogradation in the autoclaving-cooling process. The crystalline form is largely responsible for its resistance to digestive enzyme (14, 68).

#### Gelatinization profile

Gelatinization profile of corn flour and cornstarch using Rapid Visco Analyser (RVA) are shown in Table 4 and Figure 11. There is a decrease in peak viscosity in each sample after the autoclaving-cooling treatment. The peak viscosity value indicates the ability of starch granules to bind water and maintain swelling during the heating process. There was no peak viscosity (Supplementary Figure 1) in the treated sample due to the decrease of peak viscosity value after autoclaving-cooling treatment. The decrease in the peak viscosity value in the treated sample was caused by the hydrothermal treatment and high pressure during the autoclaving process, which resulted in the granule structure being destroyed thereby reducing the viscosity value (9, 38, 69). Supplementary Figure 1 also shows that the treated starch exhibits a very low viscosity which remains almost constant regardless of temperature changes. During the autoclave process, starch is hydrothermally treated under high pressure conditions. As a result, the starch is gelatinized and its granular structure is disturbed, resulting in a decrease in peak viscosity in the modified sample (38). The peak viscosity of the corn flour was smaller than the viscosity of the cornstarch. This could be due to the presence of other components such as ash, fat, and protein in the corn flour which were larger than in the cornstarch, thus affecting the gelatinization process.

**TABLE 4:** Gelatinization profile.

Parameter	Sample			
	Corn flour		Corn starch	
	Native	Treated	Native	Treated
Peak viscosity (cP)	3301.50 ± 70.00	638.50 ± 0.71	3939.50 ± 6.36	976.50 ± 10.61
Breakdown viscosity (cP)	1808.00 ± 209.30	193.50 ± 37.48	1815.00 ± 18.38	5.00 ± 5.66
Setback viscosity (cP)	3400.00 ± 103.24	592.00 ± 31.11	1879.00 ± 8.49	366.50 ± 103.94
Final viscosity (cP)	4893.50 ± 242.54	1250.00 ± 308.30	4003.50 ± 20.51	1338.00 ± 98.99
Time (min)	8.13 ± 0.00	8.30 ± 0,14	8.04 ± 0.05	11.94 ± 1.51
Temperature (°C)	74.30 ± 0.28	–	73.22 ± 0.03	–

–, undetected until 95°C.

Values expressed are mean ± standard deviation (number of replications = 2).

The final viscosity value in each sample also decreased. The final viscosity indicates the ability of the starch paste to form a gel after the heating or cooling process and the resistance of the paste to the stirring process. The gelatinization temperature of the treated sample decreased in the corn flour. The decrease in gelatinization temperature also occurred in arrowroot tuber samples that were treated with autoclaving-cooling (37). The gelatinization temperature of the treated cornstarch was not detected by RVA analysis at a maximum temperature capacity of 95°C. This indicates that the starch was gelatinized during the autoclaving-cooling process at 121°C. The gelatinization profile of the corn flour and cornstarch in this study was the same as the profile in the experiment using oat samples conducted by Shah et al. (15). The results of the RVA analysis of the treated oat samples from autoclaving-cooling two cycles showed a decrease in the value of peak viscosity, breakdown viscosity, setback viscosity, final viscosity and gelatinization temperature. Based on the gelatinization profile presented above, corn flour and cornstarch treated with autoclaving-cooling from meta-analysis results were found to be stable to heating and stirring processes. The properties of these materials are suitable to be used as ingredients for food products, especially those that require a cooking process at high temperatures, such as corn-based bakery products and canned food.

Gelatinization and retrogradation occur during the autoclaving-cooling and result in RS type 3. The retrogradation rate in cereal starch is higher than those of tuber due to higher precipitation rate of the cereal starch. Thus, the RS content increases significantly in cereal starch. Based on analysis, autoclaving-cooling on corn flour and cornstarch caused the decrease of amylose content and starch digestibility and the increase of RS content. Morphological changes were also observed after autoclaving-cooling. There was crystalline formation due to retrogradation.

## Conclusion

Meta-analysis study found that RS content significantly increased after autoclaving-cooling treatment using a sample:water ratio of 1:4, two cycles of autoclaving-cooling, and 30 min of autoclaving at 121°C. This RS increase was significant in cereal (corn, oat and rice) with corn as the type of sample that has the highest effect size value. Verification using corn flour and cornstarch showed a significant increase in RS contents and a significant decrease in starch digestibility. Treated sample also showed the pasting profile that was stable under heating and stirring. This study can provide the optimal treatment of autoclaving-cooling process to achieve a significant increase in RS content for research treatment or industrial purpose.

## Data availability statement

The raw data supporting the conclusions of this article will be made available by the authors, without undue reservation.

## Author contributions

DF: content, methodology, and schema research. RS: writing – original draft and processing data. DI: review manuscript. FA, AJ, and MA: processing data. All authors contributed to the article and approved the submitted version.

## Conflict of interest

The authors declare that the research was conducted in the absence of any commercial or financial relationships that could be construed as a potential conflict of interest.

## Publisher’s note

All claims expressed in this article are solely those of the authors and do not necessarily represent those of their affiliated organizations, or those of the publisher, the editors and the reviewers. Any product that may be evaluated in this article, or claim that may be made by its manufacturer, is not guaranteed or endorsed by the publisher.

## Supplementary material

SUPPLEMENTARY FIGURE 1 Gelatinization profile of corn flour and cornstarch.
