# Supplementary figures and images for: Verification of autoclaving-cooling treatment to increase the resistant starch contents in food starches based on meta-analysis result
Source: Front Nutr. 2022 Jul 19;9:904700. doi: 10.3389/fnut.2022.904700 (PMC9343710; doi:10.3389/fnut.2022.904700)

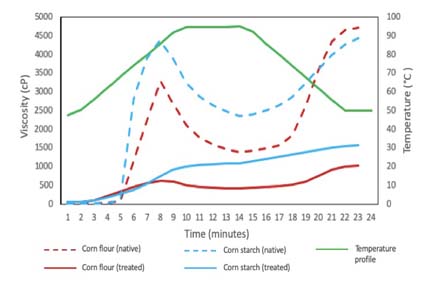

Supplement: SUPPLEMENTARY FIGURE 1 — Gelatinization profile of corn flour and cornstarch. [file Image_1.jpg]
